# 

**Published:** 1920-06-26

**Authors:** 


### Employers Subscriptions at Coventry.

A meeting of employers, convened by the Mayor, Councillor J. I. Bates, was lately held in Coventry to consider what they could do to help the Coventry and Warwickshire Hospital out of its financial difficulties. To carry on the hospital at present an increase in the annual income of £14,000 has to be raised, and if the institution is not to close down a large part or to request the City Council to take it over and place the cost of its upkeep on the rates, a large addition to the regular income must be raised under the present voluntary system.

The meeting was a representative gathering of the manufacturers in the city, and upon learning the position all present unanimously agreed that each firm in the city should be requested to contribute a sum equal to that contributed by its workpeople, with a maximum limit of 2d. per week per employee, or, in cases where this would operate unfairly, to contribute 1d. per week per employee. It was considered that this would be the most equitable arrangement. Letters to this effect are being addressed to all the firms, and a good response is anticipated. The workpeople themselves are subscribing well: in several cases they are doubling and trebling their subscriptions, and some are even proposing a flat rate of 1d. in the £.

It was noticeable that there was no suggestion in favour of the discontinuance of the voluntary system. The retailers and their assistants are next to be approached to do their share.

### Local Authorities: the Waiting-Lists.

Walsall Corporation has appointed a sub-committee to visit hospitals provided by local authorities in the surrounding districts, and to report on the accommodation which should be provided in Walsall.

### The College of Nursing (Limited by Guarantee).

### BIRMINGHAM THREE COUNTIES CENTRE.

A Members' Meeting (Miss Musson, R.R.C., in the chair) was held in the Lecture Theatre of the General Hospital, Birmingham, on Tuesday, June 22. After the Hon. Secretary had read the minutes of the previous meeting, Mrs. G. M. E. Jones (at Miss Musson's request) gave a brief *résumé* of the business transacted at the Annual Meeting of the College of Nursing on June 17. The opinion of the members present was taken as to the advisability of an annual subscription to the College, and a resolution was passed in favour of the aforesaid subscription.

Mrs. Ring (Secretary to the Birmingham Society for Equal Citizenship) then gave an instructive and interesting account of her experiences in Austria. Her suggestion that V.A.D.s and partially or untrained women should be sent out to work under Austrian matrons and sisters was not sympathetically received, the general impression being that it would create an erroneous idea in the minds of Austrian doctors as to the skill and efficiency of the British Nursing Services.

Mrs. Ring promised to bring the matter forward for the consideration of her Committee.

